# Supplementary material for: Iterative improvement in the automatic modular design of robot swarms
Source: PeerJ Comput Sci. 2020 Dec 7;6:e322. doi: 10.7717/peerj-cs.322 (PMC7924708; doi:10.7717/peerj-cs.322)
Supplement: Supplemental Information 3 [file peerj-cs-06-322-s003.zip › argos3/doc/api/standalone/a00659_source.html]

ARGoS: plugins/simulator/visualizations/qt-opengl/qtopengl\_user\_functions.h Source File


- Main Page
- Related Pages
- Namespaces
- Classes
- Files

- File List
- File Members

# plugins/simulator/visualizations/qt-opengl/qtopengl\_user\_functions.h

Go to the documentation of this file.

```
00001 
00007 #ifndef QTOPENGL_USER_FUNCTIONS_H
00008 #define QTOPENGL_USER_FUNCTIONS_H
00009 
00010 namespace argos {
00011    class CQTOpenGLUserFunctions;
00012    class CFloorEntity;
00013 }
00014 
00015 class QPainter;
00016 
00017 #include <argos3/core/utility/configuration/base_configurable_resource.h>
00018 #include <argos3/plugins/simulator/visualizations/qt-opengl/qtopengl_main_window.h>
00019 #include <argos3/plugins/simulator/visualizations/qt-opengl/qtopengl_widget.h>
00020 #include <argos3/core/utility/datatypes/color.h>
00021 #include <argos3/core/utility/math/quaternion.h>
00022 
00023 namespace argos {
00024 
00074    class CQTOpenGLUserFunctions : public CBaseConfigurableResource {
00075 
00076    public:
00077 
00081       CQTOpenGLUserFunctions();
00082 
00086       virtual ~CQTOpenGLUserFunctions();
00087 
00088       virtual void Init(TConfigurationNode& t_tree) {}
00089       virtual void Reset() {}
00090       virtual void Destroy() {}
00091 
00104       virtual void KeyPressed(QKeyEvent* pc_event);
00105 
00118       virtual void KeyReleased(QKeyEvent* pc_event);
00119 
00124       virtual void EntitySelected(CEntity& c_entity) {}
00125 
00130       virtual void EntityDeselected(CEntity& c_entity) {}
00131 
00135       CEntity* GetSelectedEntity();
00136 
00145       virtual void SelectEntity(CEntity& c_entity);
00146 
00153       virtual void DeselectEntity();
00154 
00158       virtual void Draw(CFloorEntity& c_entity) {}
00159 
00165       virtual void DrawInWorld() {}
00166 
00174       virtual void DrawOverlay(QPainter& c_painter) {}
00175 
00180       CQTOpenGLMainWindow& GetMainWindow();
00181 
00186       void SetMainWindow(CQTOpenGLMainWindow& c_main_win);
00187 
00192       CQTOpenGLWidget& GetQTOpenGLWidget();
00193 
00198       void SetColor(const CColor& c_color);
00199 
00207       void DrawPoint(const CVector3& c_position,
00208                      const CColor& c_color = CColor::RED,
00209                      Real f_diameter  = 5.0);
00210 
00222       void DrawTriangle(const CVector3& c_position,
00223                         const CQuaternion& c_orientation,
00224                         Real f_base,
00225                         Real f_height,
00226                         const CColor& c_color = CColor::RED,
00227                         const bool b_fill = true);
00228 
00238       void DrawPolygon(const CVector3& c_position,
00239                        const CQuaternion& c_orientation,
00240                        const std::vector<CVector2>& vec_points,
00241                        const CColor& c_color = CColor::RED,
00242                        const bool b_fill = true);
00243 
00255       void DrawCircle(const CVector3& c_position,
00256                       const CQuaternion& c_orientation,
00257                       Real f_radius,
00258                       const CColor& c_color = CColor::RED,
00259                       const bool b_fill = true,
00260                       GLuint un_vertices = 20);
00261 
00273       void DrawCylinder(const CVector3& c_position,
00274                         const CQuaternion& c_orientation,
00275                         Real f_radius,
00276                         Real f_height,
00277                         const CColor& c_color = CColor::RED,
00278                         GLuint un_vertices = 20);
00279 
00289       void DrawBox(const CVector3& c_position,
00290                    const CQuaternion& c_orientation,
00291                    const CVector3& c_size,
00292                    const CColor& c_color = CColor::RED);
00293 
00301       void DrawRay(const CRay3& c_ray,
00302                    const CColor& c_color = CColor::RED,
00303                    Real f_width = 1.0f);
00304 
00313       void DrawText(const CVector3& c_position,
00314                     const std::string& str_text,
00315                     const CColor& c_color = CColor::BLACK,
00316                     const QFont& c_font = QFont());
00317 
00318    protected:
00319 
00324       typedef void (CQTOpenGLUserFunctions::*TThunk)(CEntity&);
00325 
00335       template <typename USER_IMPL, typename ENTITY>
00336       void Thunk(CEntity& c_entity);
00337 
00342       class CFunctionHolder {};
00343 
00351       template <typename USER_IMPL, typename ENTITY> class CFunctionHolderImpl : public CFunctionHolder {
00352       public:
00353          typedef void (USER_IMPL::*TFunction)(ENTITY&);
00354          TFunction Function;
00355          CFunctionHolderImpl(TFunction t_function) : Function(t_function) {}
00356       };
00357 
00363       CVTable<CQTOpenGLUserFunctions, CEntity, TThunk> m_cThunks;
00364 
00369       std::vector<CFunctionHolder*> m_vecFunctionHolders;
00370 
00371    public:
00372 
00379       template <typename USER_IMPL, typename ENTITY>
00380       void RegisterUserFunction(void(USER_IMPL::*pt_function)(ENTITY&));
00381 
00386       virtual void Call(CEntity& c_entity);
00387 
00388    private:
00389 
00393       CQTOpenGLMainWindow* m_pcQTOpenGLMainWindow;
00394 
00395    };
00396 
00397    /****************************************/
00398    /****************************************/
00399 
00400    template <typename USER_IMPL, typename ENTITY>
00401    void CQTOpenGLUserFunctions::Thunk(CEntity& c_entity) {
00402       /*
00403        * When this method is called, the static type of 'this'
00404        * is CQTOpenGLUserFunctions. Since we want to call
00405        * method in USER_IMPL (subclass of CQTOpenGLUserFunctions),
00406        * we need a cast. The cast is static because we trust
00407        * the user on not doing anything stupid.
00408        * The variable cImpl can be static because the cast is necessary
00409        * only the first time this function is called.
00410        */
00411       static USER_IMPL& cImpl = static_cast<USER_IMPL&>(*this);
00412       /* Cast the argument to the right type */
00413       ENTITY& cEntity = static_cast<ENTITY&>(c_entity);
00414       /* Cast the function holder to its effective type */
00415       CFunctionHolderImpl<USER_IMPL,ENTITY>& cFunctionHolder = static_cast<CFunctionHolderImpl<USER_IMPL,ENTITY>&>(*m_vecFunctionHolders[GetTag<ENTITY,CEntity>()]);
00416       /* Call the user-defined method */
00417       (cImpl.*(cFunctionHolder.Function))(cEntity);
00418    }
00419 
00420    template <typename USER_IMPL, typename ENTITY>
00421    void CQTOpenGLUserFunctions::RegisterUserFunction(void(USER_IMPL::*pt_function)(ENTITY&)) {
00422       /* Add the thunk to the VTable */
00423       m_cThunks.Add<ENTITY>(&CQTOpenGLUserFunctions::Thunk<USER_IMPL,ENTITY>);
00424       /* Add the function holder to the vector, padding gaps with NULL pointers */
00425       size_t unIdx = GetTag<ENTITY,CEntity>();
00426       if(m_vecFunctionHolders.size() <= unIdx) {
00427          m_vecFunctionHolders.resize(unIdx+1, NULL);
00428       }
00429       m_vecFunctionHolders[unIdx] = new CFunctionHolderImpl<USER_IMPL,ENTITY>(pt_function);
00430    }
00431 
00432    /****************************************/
00433    /****************************************/
00434 
00435 }
00436 
00437 /* Definitions useful for dynamic linking of user functions */
00438 #define REGISTER_QTOPENGL_USER_FUNCTIONS(CLASSNAME, LABEL)  \
00439    REGISTER_SYMBOL(CQTOpenGLUserFunctions,                  \
00440                    CLASSNAME,                               \
00441                    LABEL,                                   \
00442                    "undefined",                             \
00443                    "undefined",                             \
00444                    "undefined",                             \
00445                    "undefined",                             \
00446                    "undefined")
00447 
00448 #endif
```

---

Generated on 10 Jul 2018 for ARGoS by 
 1.6.1
